# Experiences, Attitudes, and Needs of Users of a Pregnancy and Parenting App (Baby Buddy) During the COVID-19 Pandemic: Mixed Methods Study

**DOI:** 10.2196/23157

**Published:** 2020-12-09

**Authors:** Alexandra Rhodes, Sara Kheireddine, Andrea D Smith

**Affiliations:** 1 Department of Behavioural Science and Health Institute of Epidemiology and Health Care University College London London United Kingdom

**Keywords:** pregnancy, parenting, app, COVID-19, pregnancy support, postnatal support, perinatal, mental well-being, physical well-being, support, well-being, experience, attitude, needs

## Abstract

**Background:**

The COVID-19 pandemic has impacted the lives of expectant parents and parents of young babies, with disruptions in health care provision and loss of social support.

**Objective:**

This study investigated the impact of the COVID-19 pandemic and its associated lockdown on this population through the lens of users of the UK National Health Service–approved pregnancy and parenting smartphone app, Baby Buddy. The study aims were threefold: to gain insights into the attitudes and experiences of expectant and recent parents (with babies under 24 weeks of age) during the COVID-19 pandemic; to investigate whether Baby Buddy is meeting users’ needs during this time; and to identify ways to revise the content of Baby Buddy to better support its users now and in future.

**Methods:**

A mixed methods study design combining a web-based survey with semistructured telephone interviews among Baby Buddy users in the United Kingdom was applied. Data were collected from April 15 to mid-June 2020, corresponding to weeks 4-13 of the lockdown in the United Kingdom.

**Results:**

A total of 436 expectant (n=244, 56.0%) and recent (n=192, 44.0%) parents responded to the web-based survey, of which 79.1% (n=345) were aged 25-39 years and 17.2% (n=75) spoke English as their second language. Of the 436 respondents, 88.5% (386/436) reported increased levels of anxiety around pregnancy, birth, and being a new parent, and 58.0% (253/436) were concerned about their emotional and mental health. Of the 244 pregnant respondents, 43.4% (n=106) were concerned about their physical health. Telephone interviews with 13 pregnant women and 19 recent parents revealed similarly increased levels of anxiety due to reduced health care provision and loss of support from friends and family. Although a minority of respondents identified some positive outcomes of lockdown, such as family bonding, many telephone interviewees reported feeling isolated, disregarded, and overwhelmed. Recent parents were particularly anxious about the impact of the lockdown on their baby’s development and socialization. Many interviewees were also concerned about their physical health as a consequence of both limited access to face-to-face medical appointments and their own poorer dietary and physical activity behaviors. Across both samples, 97.0% (423/436) of respondents reported that Baby Buddy was currently helping them, with many commenting that its role was even more important given the lack of face-to-face support from health care and parenting organizations. Greater speed in updating digital content to reflect changes due to the pandemic was suggested.

**Conclusions:**

The COVID-19 pandemic has created heightened anxiety and stress among expectant parents and those with a young baby, and for many, lockdown has had an adverse impact on their physical and mental well-being. With reductions in health care and social support, expectant and new parents are increasingly relying on web-based resources. As a free, evidence-based app, Baby Buddy is well positioned to meet this need. The app could support its users even more by actively directing them to the wealth of existing content relevant to their concerns and by adding content to give users the knowledge and confidence to meet new challenges.

## Introduction

The COVID-19 pandemic has impacted people’s lives in ways that few could have predicted. With health care services channeling resources to patients with COVID-19 and face-to-face contacts being reduced to limit the risk of contagion, antenatal, birth and postnatal care have all been affected. Moreover, due to the COVID-19 pandemic and its associated lockdown, expectant parents and parents of young babies have lost their usual support networks.

In recent years, the use of pregnancy and parenting apps has become widespread [1,2]. A 2016 study in Australia found that three-quarters of a sample (n=410) of pregnant women and women who had had a baby in the last three years had used a pregnancy app, and over half had used a parenting app [3]. In terms of the people who use pregnancy apps, research suggests that women who are pregnant for the first time and have owned a smartphone for longer have higher uptake [4], while women with lower income and nonnative speakers of the app language have lower uptake [2]. Access to information on pregnancy health and fetal development has been reported to be the primary reason for using a pregnancy app, along with access to information on nutrition [2,5]. Although data on the use of pregnancy and parenting apps in the United Kingdom are not available, smartphone ownership is reported to be in excess of 90% among UK women of childbearing age [6].

Launched in the United Kingdom in 2014, Baby Buddy is a pregnancy and parenting app approved by the National Health Service (NHS) that is designed to support expectant and new parents through pregnancy and the firs*t* 24 weeks of parenthood and to reduce inequalities [7]. Baby Buddy was developed by taking a proportionate universalism approach: it was designed and developed for use by all UK parents but was created to be particularly relevant and engaging for parents from communities whose voices are seldom heard and whose children are at higher risk of poorer outcomes [8]. The app is free to download and is available in all app stores. In addition, Best Beginnings, the charity behind Baby Buddy, works with NHS trusts and local authorities to embed the app into maternity care pathways. Baby Buddy provides trusted, evidence-based information and self-care tools to help expectant and new parents build their knowledge and confidence and effect positive behavior changes. Its content and functionality were co-created with parents and professional health organizations. With low literacy level requirements and extensive video content (over 300 short video clips of parents sharing their own stories and professionals sharing advice), Baby Buddy ensures inclusivity and is easily accessible to people not in education, training, or employment and those whose first language is not English (see Multimedia Appendix 1 for more details about the app). Contrary to other pregnancy apps [2], Baby Buddy data show disproportionate usage by young women (under 25 years of age), women from Black, Asian, and minority ethnic communities, and people who speak English as a second language (ESL) [9].

As a virtual resource, Baby Buddy has been able to continue to provide support to its users during the pandemic. However, Best Beginnings was quick to recognize that the needs of Baby Buddy users and the role the app plays for them might have shifted during this period. Therefore, the decision was made to conduct a service evaluation study to identify how Baby Buddy is currently meeting its users’ needs and how to increase the value of the app to its users during this time. With a new version of Baby Buddy due to launch in 2021, the findings from this research could also feed into the future development of the app. This study acted as the precursor to a UK population-wide web-based survey exploring the experiences of expectant parents and parents of babies under 2 years of age, Babies in Lockdown [10].

The aims of this study were threefold: first, to gain insights into the attitudes and experiences of expectant parents and parents of very young babies during the COVID-19 pandemic; second, to investigate whether Baby Buddy is meeting the needs of its users during this time; and third, to identify ways in which the content of Baby Buddy can be added to or updated to better support its users both now and in the future.

## Methods

### Ethical Approval

As a service evaluation study, this project was exempt from University College London (UCL) ethical approval. However, procedures for informing respondents about the research and how their data would be handled, maintaining their anonymity, and obtaining respondent consent complied with the General Data Protection Regulation (GDPR) and were consistent with standards demanded by UCL ethics.

Given the potential mental fragility of respondents caused by the COVID-19 pandemic, a strategy for providing follow-up support was planned, including links to support within the app (eg, the 24/7 Baby Buddy Crisis Messenger clinically supervised text service) and telephone and web-based support from other charitable organizations.

### Study Design

The study used a mixed methods approach comprising an anonymous web-based survey and a series of semistructured telephone interviews among a sample of survey respondents who expressed willingness to take part in further research.

The web-based survey was designed to capture the impact of COVID-19 on the respondents’ views and experiences of being pregnant or parenting a young baby, mood, levels of anxiety, key concerns, and additional worries; the perceived impact of the pandemic on their baby; contact with health care professionals and perinatal support; and their use and views of Baby Buddy. The survey was hosted on SurveyMonkey, with collection settings to anonymize responses and disallow multiple responses from the same IP address. The survey comprised 12 questions (over 6 pages), all but 3 of which were mandatory and 8 of which included free text boxes for respondents to record their opinions in their own words. The questions were worded to reflect the status of the respondent (ie, pregnant, gave birth within the last 6 months, or partner of either) established in question 1. Minimal demographic information (age, ESL, and region) was collected to reduce respondent burden. The survey was piloted with expectant and new parents (n=4) and professionals working in the perinatal field (n=4). Data collection occurred between April 15 and May 31, 2020.

The topic guide for the telephone interviews was designed by AR and approved by Best Beginnings (see Multimedia Appendix 2). All interviews were conducted by AR, an experienced qualitative researcher with many years of experience conducting sensitive research, and SK, who received training and guidance on interviewing approaches and techniques from AR. Telephone interviews took place from April 29 to June 18, 2020.

### Sample and Recruitment Strategy

A convenience sample of Baby Buddy app users was recruited via the app. A notification and link to the survey was incorporated into many pieces of daily information within the Today’s Information feature in the app from April 15 to May 31, 2020 (see Multimedia Appendix 3 for the recruitment message). The Today’s Information feature delivers a new piece of gestational stage–appropriate information each morning to the user’s home screen. The link was added to every seventh piece of daily information to ensure that it would be delivered to all users once over the course of a week irrespective of their gestational stage or baby age. The link took potential respondents to a survey information page that outlined the anonymous nature of the survey and how the data would be reported; the page also provided a link to the Best Beginnings privacy statement as well as an email address for queries. Participants’ consent was sought before they proceeded to the survey.

Telephone interview respondents were selected from the list of those who agreed to participate in further research (requested at the end of the web-based survey) and had provided contact details. Selection was initially random but was later based on age, postcode, and ESL to ensure a representative sample. An a priori decision was made to conduct a minimum of 20 interviews, after which AR and SK would decide whether the saturation point had been reached and, if not, among which respondent types further investigation was required.

Neither the web-based survey nor the telephone interview respondents were offered any incentives.

### Data Analysis

Quantitative data analyses were undertaken in SPSS version 26 (IBM Corporation). Pearson chi-square tests were used to determine whether responses varied according to age or ESL. Qualitative data (telephone interviews and comments from the free text boxes) was analyzed thematically [11]. The 5-stage analysis process was conducted by hand. First, AR and SK listened to and transcribed their own interviews as well as 30% of each other’s interviews. Second, AR developed an initial coding framework (see Multimedia Appendix 4) based on key topics from the interview guide and free text boxes as well as additional, unanticipated topics that emerged from the first five interviews [12]. Third, AR and SK coded their own and 30% of each other’s interview transcripts and AR coded the free text comments from the survey. The coding framework continued to be refined throughout the coding process, with any discrepancies between AR and SK over codes or coding definitions being resolved through discussion. Fourth, emerging themes were identified, and AR and SK agreed on the final themes through an iterative process of discussion, refinement, and development. Fifth, AR and SK revisited the transcripts to confirm the legitimacy of the final themes. AS verified the coding and theme development process for accuracy and appropriateness.

## Results

### Participant Characteristics

#### Web-Based Survey

A total of 543 Baby Buddy app users opened the survey, and 436 (80.7%) completed it; however, 23 users (4.2%) declined to answer the demographic questions, of whom 14 (2.6) also did not answer the last three survey questions. The characteristics of the final sample are shown in Table 1. Of the sample, 55.9% respondents were pregnant (244/436) and 44.0% were recent parents (192/436). Only 2.1% (9/436) of the sample were partners of pregnant women or women who had recently given birth. The majority of the sample was aged between 25 and 39 years (345/436, 79.1%) and spoke English as their first language (361/436, 82.8%).

**Table 1:** Descriptive characteristics of the web-based survey respondents by age group (N=436), n (%).

Characteristic		Pregnant	Partner pregnant	Given birth in last 6 months	Partner given birth in last 6 months	Total
**Age (years)**						
	<21	12 (2.8)	0 (0)	3 (0.7)	0 (0)	15 (3.4)
	21-24	15 (3.4)	0 (0)	8 (1.8)	0 (0)	23 (5.3)
	25-29	64 (14.7)	0 (0)	40 (9.2)	1 (0.2)	105 (24.1)
	30-34	76 (17.4)	1 (0.2)	83 (19.0)	2 (0.5)	162 (37.1)
	35-39	41 (9.4)	1 (0.2)	35 (8.0)	1 (0.2)	78 (17.9)
	40-44	12 (2.8)	0 (0)	14 (3.2)	0 (0)	26 (6.0)
	≥45	2 (0.5)	1 (0,2)	1 (0.2)	0 (0)	4 (0.9)
	Did not answer	17 (3.9)	2 (0.5)	4 (0.9)	0 (0)	23 (5.3)
	Total	239 (54.8)	5 (1.1)	188 (43.1)	4 (0.9)	436 (100)
ESL^a^		44 (10.1)	1 (0.2)	28 (6.4)	2 (0.5)	75 (17.2)

^a^ESL: English as a second language.

#### Telephone Interviews

A total of 32 telephone interviews were conducted (AR, n=19, 59%; SK, n=13, 41%). With the exception of one interview that lasted 12 minutes, the interviews lasted between 21 and 55 minutes, averaging 30 minutes (median interview length 28 minutes). Table 2 summarizes the telephone interview sample characteristics. The majority of respondents were women who were married or cohabiting. Of these 32 respondents, 14 (44%) were pregnant women (between 8 and 38 weeks), 2 of whom (14%) had another child or children. The remaining 18 respondents (56%) had a baby under 6 months of age (between 3 and 24 weeks), 2 of whom (11%) had another child and 1 of whom (6%) was a father. Of the 18 postnatal respondents, 2 (11%) had given birth during the lockdown. The majority of the sample was of White British ethnicity (21/32, 66%). Of the 32 respondents, 6 (19%) were ESL respondents.

**Table 2:** Descriptive characteristics of the telephone interviewees by age group (N=32), n (%).

Age (years)	Pregnant (n=13)			Postnatal (n=19)		
	White British	White other	Black, Asian, minority ethnicity	White British	White other	Black, Asian, minority ethnicity
21-24	1 (3)	1 (3)	1 (3)	0 (0)	0 (0)	0 (0)
25-29	0 (0)	0 (0)	2 (6)	2 (6)	1 (3)	1 (3)
30-34	2 (6)	1 (3)	0 (0)	6 (19)	2 (6)	1 (3)
35-39	2 (6)	0 (0)	1 (3)	3 (9)	0 (0)	0 (0)
40-44	2 (6)	0 (0)	0 (0)	2 (6)	0 (0)	0 (0)
≥45	0 (0)	0 (0)	0 (0)	1 (3)	0 (0)	0 (0)
Total	7 (22)	2 (6)	4 (13)	14 (44)	3 (9)	2 (6)

### Overview of Quantitative and Qualitative Findings

Given the alignment of the quantitative and qualitative findings and to create a holistic picture, the results of the web-based survey and telephone interviews are presented together under the four main themes that emerged from the analysis of the qualitative data (see Multimedia Appendix 5 for theme definitions and examples). The first three themes are interconnected and consider the impact of COVID-19 on expectant parents and parents of very young babies. The first theme explores how the pandemic has resulted in increased levels of anxiety and stress, identifying three underlying causes: current disruption, future uncertainties, and fear of contracting COVID-19. The second and third themes expand on these underlying causes, focusing on the aspects of life during the pandemic that have most affected pregnant and postnatal women—reduced levels of support and life under lockdown. The final theme considers the use of Baby Buddy during the pandemic and respondents’ ideas for additional content.

No statistically significant differences were detected according to age or ESL.

### Increased Levels of Anxiety and Stress

Of the 436 web-based survey respondents, 88.5% (n=386) reported that the pandemic had increased their levels of anxiety around pregnancy, birth, and being a new parent, with 42.8% (n=187) claiming the pandemic had made them a lot more anxious (see Table 3). The negative impact of the pandemic on mental well-being was also reflected in the web-based survey responses to the question “Please can you give us three words to describe your mood over the past 5 days,” with *anxious* appearing alongside *happy* and *tired* as the three most commonly used words. In response to the question “What are your main concerns right now?” which included 11 options as well as “none of the above” and “other” (see Multimedia Appendix 6), over 60% of respondents identified their own emotional and mental health to be a main concern. For the 244 pregnant respondents, this was the highest scoring main concern at that moment (61.3%, 144/235), while for postnatal respondents, it was second (58.0%, 109/188) to concerns about their baby’s health (63.3%, 119/188). The qualitative findings indicated a range of negative emotional states in addition to anxiety and stress, including loneliness, irritability, sadness, and depression.

I am a lot more tense and anxious some days more than others and then I am very irritable. I cannot go out much and don't really have motivation to talk to people.

Free text

I have had days when I break down and cry….my entire day is lying on the sofa or in bed doing absolutely nothing.

Pregnant 21 weeks, age 20-24 years, Black, Asian, minority ethnicity

I have started to struggle in the last two weeks…. I had had a fight with my boyfriend because I was so tired

Baby 16 weeks, age 30-34 years, White other

I have become lazy – I can’t be bothered…lethargic. I don’t feel hungry because I am not moving enough…I can’t be bothered with cooking or cleaning now….the boys have lost their appetite – one boy is losing weight….they stay in their PJs all day, say there is no point in brushing their teeth as no one is going to smell their breath….the boys are worried that when I go to hospital I am going to die.

Pregnant 34 weeks, age 25-29 years, Black, Asian, minority ethnicity

**Table 3:** Levels of anxiety about pregnancy and birth (pregnant) and having a new baby (postnatal) during weeks 1-2 and weeks 3-4 (N=436), n (%).

Anxiety level^a^	Pregnant			Postnatal		
	Overall (n=244)	Weeks 1-2 (April 15-29, n=116)	Weeks 3-4 (May 6-20, n=75)	Overall (n=192)	Weeks 1-2 (April 15-22, n=74)	Weeks 3-4 (May 6-20, n=65)
A lot more anxious	108 (44.2)	56 (48.3)	31 (41.3)	79 (41.1)	21 (28.4)	32 (49.2)
A little more anxious	111 (45.5)	50 (43.1)	33 (44)	88 (45.8)	43 (58.1)	25 (38.5)
No different	23 (9.4)	10 (8.6)	9 (12)	18 (9.3)	8 (10.8)	6 (9.2)
A little less anxious	1 (0.5)	0 (0)	1 (1.3)	7 (3.6)	2 (2.7)	2 (3.1)
A lot less anxious	1 (0.5)	0 (0)	1 (1.3)	0 (0)	0 (0)	0 (0)

^a^Evaluated with a 5-point Likert scale. Questions asked: “How has coronavirus made you feel about pregnancy and birth?” (pregnant) and “How has coronavirus made you feel about being a new parent or supporting a new parent?” (postnatal).

Not surprisingly, people already suffering from mental health issues had been hit particularly hard by the pandemic.

I have struggled with postnatal depression, OCD [obsessive-compulsive disorder] and anxiety. The disruption to normal life has affected the progress I had been making.

Free text

A small minority of respondents painted a more positive picture of their state of mind, finding their home-based life relaxing and less stressful than “normal” life.

Not having to go out the house as much has made me more relaxed.

Free text

The qualitative findings indicated three main causes of these negative emotional states: fear of contracting COVID-19, current disruption, and future uncertainties. The contribution of these causes to anxiety and stress levels appeared to change over the course of the research; current disruption and fear of contracting the virus were responsible for raised anxiety and stress levels during the early weeks (April), but future uncertainties showed more impact later (May). Similar shifts were apparent in the web-based survey, which showed levels of anxiety decreasing slightly over time among pregnant respondents but increasing slightly over time among postnatal respondents (see Table 3). Possible explanations for this finding are discussed in the following sections.

#### Fear of Contracting COVID-19

During April, when the pandemic was peeking in the United Kingdom, anxiety over contracting COVID-19 was high. Pregnant women expressed anxiety over the impact their contracting the virus might have on their unborn child. Those close to delivery were particularly anxious about contracting COVID-19 in hospital when giving birth. Indeed, over two-thirds of the pregnant web-based survey respondents (152/235, 64.7%) said that staying safe when giving birth was worrying them more than normal at the moment, and over half (125/235, 53.2%) were worried about staying safe at antenatal appointments (see Multimedia Appendix 7).

I’m scared of the unknown- how much does it affect pregnant women and unborn babies, will my new-born be at huge risk?

Free text

While the most common concern for postnatal respondents was their baby’s health, qualitative insights showed that respondents’ concerns did not revolve around their baby contracting the virus but around the potential impact on their baby should they themselves contract COVID-19, such as whether they would still be able to breastfeed and who would look after their baby.

I don’t want my baby to catch anything while he is so little. I don’t want to catch coronavirus and leave my child without a parent.

Free text

Qualitative findings suggested that for many respondents, fear of contracting COVID-19 diminished during May, perhaps because death rates fell and respondents felt safer because lockdown and social distancing measures were well-established.

I am happy with lockdown as I feel my baby and I are protected.

Free text

The exception was respondents whose risk of contracting COVID-19 was above average, including respondents who were of Black, Asian, or minority ethnicity, those with underlying health conditions, and those whose partners were health care workers.

It’s a worrying time to be health care professionals and being pregnant. I worry that my husband will get coronavirus and infect me and that could result in FGR [fetal growth restriction] or worse.

Free text

#### Current Disruption

During the first weeks of the research (weeks 4-6 of lockdown), the respondents’ focus appeared to be on current disruptions caused by the pandemic and proximate uncertainties such as accessibility to food and medicines, job insecurity, and financial worries. Current disruption seemed to be greater for pregnant respondents for a number of reasons. For some pregnant women, there was considerable confusion as to whether they should be going to work if employers were not necessarily aware of or adhering to government advice.

I’m a community care worker. I’ve had problems with work as they won’t pay me sick pay and they haven’t fired me, they just aren’t giving me any shifts so I’ve lost my income.

Pregnant 8 weeks, age 21-24 years, White British

Many pregnant respondents stated that the changes to their antenatal care schedule were unsettling, with some appointments being cancelled. Some women struggled to make contact with their midwives, especially in the early days of lockdown. For those whose due date was imminent, disruption of birth plans was a major source of anxiety, with the single greatest fear being having to go through labor and give birth without their partner. However, later survey responses and interviews suggested that some of these fears had subsided as reports circulated of good birth experiences, quiet labor wards, and partners being allowed to be present. Although 69.0% (78/113) of pregnant respondents said they were more worried than usual about staying safe when giving birth in the first two weeks of the survey, this proportion dropped to 57.9% (22/38) in the last two weeks of the survey (see Multimedia Appendix 7).

#### Future Uncertainties

Over the course of the research, a shift appeared to occur from focusing on current disruption to future uncertainties. Postnatal respondents were particularly anxious about the longer-term impact of the pandemic, such as limited access to support services to monitor their baby’s development, the impact of lockdown on their baby’s socialization, and how both they and their baby would cope with returning to work. Whereas 42% (78/187) of all respondents said they were more worried than usual about caring for a new baby under the "stay at home advice" in the first two weeks of the survey, this had risen to 55% (44/80) in the survey’s last two weeks (see Multimedia Appendix 7).

Worried about the world – thinking ahead how’s it going to be – a massive change that we are all going to have to go through – it’s quite an anxious time really.

Baby 3 months, age 30-34 years, Black, Asian, minority ethnicity

### Emotional and Physical Impact of Reduced Support

Free text comments from the web-based survey and telephone interviews showed that the impact of loss of support on the respondents’ mental and physical well-being was substantial. Several sources of support were identified by respondents: family and friends, health care professionals, and government.

#### Support from Family and Friends

Although modern technology enables video calls with family and friends, the respondents, especially those with young babies, felt that they were suffering considerably from the loss of face-to-face interaction. Without antenatal and postnatal groups and visits to family and friends, respondents felt that they were missing out on opportunities to share experiences and learn from others. Many reported increased usage of digital pregnancy and parenting resources, and some had found online support groups and classes. In practical terms, the loss of informal childcare support (such as grandparents babysitting) meant that mothers no longer had time to themselves for mental revival or physical exercise. Several postnatal respondents remarked that their intended postpartum fitness regime had been put on hold as a consequence of having no help with childcare.

A frequently voiced concern for postnatal respondents was the stress of 24/7 parenting. They were not only struggling with the strain of having no time to themselves but were also worrying about having to be more self-reliant and responsible for monitoring their baby’s health and development. This impact was particularly magnified for those whose partners were working long hours at home or out of the home, as well as for single mothers.

My partner is working from home, so I am trying to keep him (baby) quiet. I’d just like a break. He is literally with me 100% of the time.

Baby 6 months, age 35-39 years, White British

For many, the loss of face-to-face interaction with family and friends created feelings of isolation and loneliness. For those with pre-existing mental health issues, this could be particularly challenging.

My maternity leave has not been what I had hoped. I have not been able to access help with the baby from family or friends because of social distancing. I have not had the benefits of meeting up with other mums and have felt isolated and very lonely at times.

Free text

#### Support from Health Care Professionals

The survey findings showed that for pregnant respondents, there had been an equal number of face-to-face and telephone midwife appointments (face-to-face: 134/235, 57.0%, vs telephone: 131/235, 55.7%). For postnatal respondents, health visitor appointments had been largely by telephone (telephone: 107/188, 56.9%, vs face-to-face: 24/188, 12.8%). Over 53% of pregnant respondents (125/235, 53.2%) and postnatal respondents (110/188, 58.5%) reported that they were worrying more than usual about being able to see their health care professional if they needed to (see Multimedia Appendix 7). Many respondents reported the negative impact of reduced support from health care professionals, with face-to-face antenatal and postnatal appointments being replaced with telephone appointments or simply being cancelled.

#### Pregnant Respondents

In the telephone interviews and free text sections of the survey, pregnant respondents often expressed their concern and disappointment at receiving what they regarded as suboptimal antenatal care. Although some respondents pointed out that telephone appointments reduced their risk of contracting COVID-19, many worried about the aspects of care they might be missing. Indeed, a common sentiment was that of being disregarded or sidelined.

My antenatal care is now much more rushed and stressful. There is no time to talk to the midwife and it very much feels like a quick process to check my urine and blood pressure only. This makes me feel less connected and more anxious.

Free text

I’ve had days when I break down and cry. We aren’t getting as much support from the midwives. I am out of vitamins and the children's centres are closed. I’ve had 6 different midwives and when I call them I never get a reply, just a message, sometimes saying they are no longer working.

Pregnant 21 weeks, age 21-24 years, Black, Asian, minority ethnicity

I want to hear my baby’s heartbeat – a phone call’s not the same.

Pregnant 7 weeks, age 35-39 years, White other

While there was no evidence that pregnant women themselves chose to avoid antenatal appointments, many talked about their initial reluctance to visit a hospital, especially since their partner was unable to accompany them. However, their experiences had generally been positive, and separate areas or entrances for antenatal patients had reassured respondents.

In most instances, reduced health care professional support did not appear to have impacted the safety or health of the pregnant women. However, in a number of more concerning cases, the absence of face-to-face consultation had impacted the respondents both mentally and physically.

I had a bladder infection. They were like you can’t come in to the doctor’s, so everything was by phone and they didn’t test it and then I had to make a choice, if I wanted to just take the lower tablets which would cover the bladder infection but not kidney, or the kidney ones but the kidney ones had a chance of miscarriage … stuff like that I find quite scary.

Pregnant, 13 weeks, age 30-34 years, White British

#### Postnatal Respondents

Qualitative findings suggested that the reduced support from health care professionals had a greater impact on postnatal respondents. A widespread lack of face-to-face appointments with health visitors and general practitioners (GPs) meant that many respondents were worried about their baby’s health and development.

They were worried that he wasn’t putting on weight…he was referred to the GP for acid reflux but we only had a telephone consultation…. the health visitor isn’t coming any more. When I asked what I could do, she said check whether he is growing out of his baby grows [all-in-one sleep suits].

Baby 8 weeks, age 40-44 years, White British

My baby was on a feeding plan but I can’t have her weighed anymore and the doctor has diagnosed reflux over the phone but the medication we’ve tried isn’t working. I feel quite tearful and less supported than at first.

Free text

Although some respondents had been able to speak to their health visitor on the telephone, others reported having no health visitor service at all. Concerns about the baby’s weight gain were frequently voiced, as were breastfeeding issues by those who had given birth just before or during lockdown.

I was struggling breastfeeding. I would have gone to breastfeeding group, but that’s been cancelled…. I was in pain and I felt let down, but I felt really guilty asking for help.

Baby 7 weeks, age 30-34 years, White British

Feeding is a challenge at the moment….it’s been painful. I was engorged and he was crying, and I was upset that he was upset, so my partner went to get some formula, but Tesco’s isn’*t* 24 hours anymore.

Baby 7 weeks, age 30-34 years, White British

Several respondents talked about their threshold for seeking medical or developmental advice for their baby being higher, both as a result of their concerns about burdening an already stressed health service and the safety of visiting a GP surgery or hospital. The resulting greater self-reliance in monitoring and diagnosing issues with their baby was worrying and stressful.

You can’t get things now – you can’t get a thermometer…. if you are a bit anxious because the baby is unwell that ramps up 20 times because you can’t do anything.

Baby 7 weeks, age 30-34 years, White British

No-one has seen him in 2 months. What if there’s something he’s doing that’s not right?

Baby 5 months, age 25-29 years, White British

A small but meaningful number of respondents had experienced postponed or cancelled hospital appointments for themselves or their baby, which had caused substantial anxiety.

He’s got a cows’ milk allergy and kept being sick…with what’s going on, I think he’s been forgotten – I feel as if it’s been left. We were supposed to have an appointment with the pediatrician in March, they were going to weigh him, but I still haven’t had my appointment….I think its affected my mental health a bit. I feel overwhelmed.

Baby 6 months, age 35-39 years, White British

I’ve been recommended to attend the ‘birth afterthoughts’ service to deal with my feelings over difficult birth but am wary of attending any hospital appts that are not absolutely necessary. Similarly baby is supposed to be referred to paediatric cardiac specialist- I’ve heard nothing...and hesitant to chase for same reasons.

Free text

Many postnatal respondents questioned the cancellation of 6-week postnatal checks. Although for most respondents, this had little impact beyond reinforcing the sense of being disregarded by the NHS, a small number of respondents felt that their recovery had been hindered by not being able to see their GP.

I had an episiotomy and I’m still in pain 7 weeks later. The doctor said no six-week checks … eventually the doctor gave me a call but I haven’t had an examination. I feel as if I have been short changed.

Baby 7 weeks, age 30-34 years, White British

I didn’t get the aftercare I was expecting and I’m in agony but not able to get real help from the NHS. Just feels like I had my baby and then abandoned.

Free text

One exception to postnatal respondents’ general dissatisfaction with health care services was baby vaccinations. Respondents reported positive experiences of visiting GP surgeries, remarking on reassuringly high levels of hygiene and social distancing precautions.

#### Support from Government

A lack of clear guidelines from the government led some respondents to feel that they were a forgotten sector of the population. Although initial guidelines had suggested that pregnant women should shield, there was confusion as to whether this meant staying indoors or being allowed out to exercise and whether it was still applicable (by May) or had been superseded by new advice. Postnatal respondents were not aware of any advice directed at them as parents of young babies, and many were critical of its absence.

I just feel like new moms and parents have just been completely left out of the Government’s mindset and their exit strategy really.

Baby 3 months, age 30-34 years, White British

I don’t think I am being supported. The Government should be giving us more information, like where do you go to get your baby weighed…there were 8 weeks when she wasn’t weighed – quite a worry really.

Baby 12 weeks, age 30-34 years, White British

As talk of easing lockdown restrictions began (mid-May), postnatal respondents were particularly frustrated by the lack of consideration given to their circumstances.

I would like someone to be representing our needs to politicians so that priority is given to measures like “bubbles” that would help new families SO much rather than people being allowed to have a cleaner come!

Free text

### Life Under Lockdown

Unsurprisingly, lockdown had an immense impact on all aspects of the respondents’ lives. Within the context of pregnancy and parenting, four subthemes emerged from the qualitative analysis: missing out, lifestyle disruption, couple relationships, and impact on baby.

#### Missing Out

The greatest disappointment for pregnant respondents appeared to be that their partners were missing out on antenatal appointments. Women worried that less involvement might adversely affect their partner’s bonding with the baby. Missing out on antenatal groups was also disappointing for women, although some were attending existing groups on the web or had found new online support groups or forums to compensate.

We’ve looked at the videos (on Baby Buddy) of the baby moving in the womb. It’s good for my husband as he can’t go to my scans. He hasn’t even heard the heartbeat …. I worry he isn’t bonding with the bump.

Pregnant 21 weeks, age 21-24 years, Black, Asian, minority ethnicity

The sense of missing out was expressed much more strongly by postnatal respondents who felt that they were being deprived of all the fun activities they had looked forward to doing during maternity leave, such as play dates, baby classes, and seeing more of family and friends. Some respondents thought a UK-wide extension to maternity leave was needed, while those who were able to extend their maternity leave were considering doing so.

I’m due to go back in November, but I am thinking of taking the full 12 months as I haven’t been able to do stuff with her.

Baby 3 months, age 30-34 years, Black, Asian, minority ethnicity

A small minority expressed more positive views of the benefits of a quieter life.

No pressure to go to groups, no need to go places at specific times, no stress of public feeding or changing nappies in public toilets. He has 100% of my attention and is very happy and settled.

Free text

#### Lifestyle Disruption

Both pregnant and postnatal respondents talked about disruption to routine adversely affecting their physical health. Their physical health was the third (106/235, 45.1%) and fourth (65/188, 34.6%) most highly rated concern at the moment (see Multimedia Appendix 6) for pregnant and postnatal respondents, respectively. Most people were getting far less exercise than normal, as work and activities outside the home had ceased and many were reluctant to go outside at all due to fear of contracting the virus. This was particularly true for those living in urban areas, where it was difficult to conform to social distancing rules.

I don’t get out of the house for days, weeks on end….there are too many people outside….it’s getting difficult walking from room to room.

Pregnant 34 weeks, age 25-29 years, Black, Asian, minority ethnicity

We’re not getting out much – we are super cautious because of the hole in her heart….so many people around it wouldn’t have been possible to do any distancing.

Baby 16 weeks, age 30-34 years, White British

At the time of the research, the government guideline was to go out to exercise only once per day, and many parents were choosing to take their baby out over exercising themselves. For some respondents, the lack of physical activity had become self-perpetuating, with energy levels decreasing to a concerningly low level.

I hadn’t been out (from mid-March) until the end of April. I went for a very short walk – I was really tired.

Baby 4 months, age 30-34 years, White other

Similarly, many respondents talked about changes to their diet. On the positive side, some respondents reported doing more home cooking during lockdown, although they were more likely to bake than cook nutritious meals. However, 35.3% (83/235) of pregnant respondents and 28.2% (53/188) of postpartum respondents were concerned about healthy eating. A reduction in consumption of fruit and vegetables was reported widely in the telephone interviews, as many respondents were attempting to visit shops as infrequently as possible and delegating grocery shopping to their partner. Reports of more frequent snacking and treat-eating due to boredom, misery, or simply a lack of other treat options were widespread.

Physically I am a wreck. My back hurts, I never do any exercise as my baby doesn’t sleep during the day. My ligaments hurt, my breasts hurt. I want to lose some weight, but I am stress eating – I’m not hungry I just eat because I want something sweet.

Baby 16 weeks, age 30-34 years, White other

#### Couple Relationships

Here, experiences were divisive; many respondents with a partner working from home really appreciated the additional time spent together. Postnatal respondents pointed out the practical benefits of sharing childcare and household tasks as well as the emotional benefits of the greater opportunity to bond as a family.

My baby is having the best start to life! Full undivided access to her mummy and her daddy!! For 2 months!

Free text

However, for a small number of respondents, lockdown had created tensions within the couple’s relationship. Those whose partners were working hard, either from home or outside, could find themselves resenting their partner’s seemingly “normal” lives when they were feeling isolated, alone, and bored. Respondents also talked about arguments about levels of hygiene and adherence to lockdown guidelines; this seemed more prevalent in couples in which the partner was working outside the home.

There have been a couple of occasions where there have been cross words…. I am sat in this house all day alone ….with the hours he is out of the house I don’t have another adult to speak to – it’s made it a bit more isolating.

Baby 7 weeks, age 30-34 years, White British

#### Impact on Baby

Less than half of pregnant respondents (47%, 89/190) felt that the changes that the pandemic was causing in their lives were affecting their baby. Several telephone interviewees expressed concern that their stress might transfer to their baby in the womb and adversely affect its development. In contrast, over three-quarters of postnatal respondents (76%, 118/155) felt that the changes were affecting their baby, and this high level of concern was voiced strongly by telephone interviewees. They worried that their baby was missing out on interactions with other people, experiences, and activities, which would have a detrimental impact on the baby’s development and socialization. Respondents frequently expressed concerns that their baby might forget their grandparents and other family members or become clingy and reluctant to interact with others.

My baby has been unable to meet family and I am anxious that she will be fearful of others when it comes to the time she needs to go to nursery.

Free text

Lockdown had also affected some babies’ routines, with changes in sleeping patterns reported most frequently. Although some postnatal respondents viewed lockdown to be an ideal time to develop new routines, others found it challenging to cope with the changes and felt that their baby had become more unsettled and cried more frequently.

The only way I used to be able to get him to nap at lunch time was to go for a walk in the buggy … I was unable to go out at all at the start because he had a temperature so I couldn’t get him to sleep so there were about 3 weeks that were kind of hellish.

Baby 6 months, age 35-39 years, White British

### Use of the Baby Buddy App During the Pandemic

While the majority of survey and telephone interview respondents felt they were using the Baby Buddy app about the same amount during the pandemic (pregnant: 70.5%, 172/244; postnatal: 74.5%, 143/192), nearly a quarter of the survey respondents said they were using the app more (pregnant 25%, 61/244; postnatal 19.8%, 38/192). Comments from the free text boxes and telephone interviewees showed that many women were finding Baby Buddy to be particularly valuable in the absence of support from health care professionals and baby groups.

I have had very little contact with my midwife and I really like how much information I can get from the app given that I can’t seem to get any support from anywhere else.

Free text

Without groups to go to I’ve found having these apps to be even more useful and helpful.

Free text

I have enjoyed using Baby Buddy throughout pregnancy and these early months and find it even more reassuring now that external input is so limited.

Baby 7 weeks, age 30-34 years, White British

I look forward to the little bit of advice every day. It is written in such a non-judgemental way, encouraging but not patronising. I feel like it’s giving me some of the general knowledge I’d be picking up from other mums at baby groups.

Free text

Table 4 summarizes the ways in which Baby Buddy helped its users, with 90.0% (392/436) of respondents identifying at least one way in which the app was helping them during the pandemic. Access to reliable information was cited by 82.6% (360/436) of survey respondents, and telephone interviewees frequently remarked that the main strength of the app was the provision of trustworthy and easy-to-understand information. Most interview respondents had either been recommended Baby Buddy by their midwife or had seen a poster or leaflet for the app at an antenatal appointment. Therefore, they felt confident that the app met with NHS approval and that its content would be accurate and trustworthy.

Because it’s linked to the NHS it’s not like googling.

Baby 3 months, age 30-34 years, Black, Asian, minority ethnicity

Reliable, trustworthy and ad-free source of information. Great videos.

Free text

**Table 4:** Proportions of respondents’ answers to the question “How is Baby Buddy helping you at the moment? (Tick all that apply to you)” (N=436), n (%).

Type of help	Pregnant (n=244)	Postnatal (n=192)	Total (N=436)
Giving me access to reliable information	193 (79.1)	167 (87.0)	360 (82.6)
Helping me bond with my baby	60 (24.6)	64 (33.3)	124 (28.4)
Helping me with my emotional and mental health	50 (20.5)	53 (27.6)	103 (23.6)
Helping me with my physical health	26 (10.7)	27 (14.1)	53 (12.2)
Helping me with my relationships	19 (7.8)	20 (10.4)	39 (8.9)
Baby Buddy isn’t helping me	31 (12.7)	13 (6.8)	44 (10.1)
Helping me in other ways	24 (9.8)	23 (12.0)	46 (10.6)

Of the respondents who reported which Baby Buddy features they were finding most helpful at the moment, 97% (377/436) identified Today’s Information (see Multimedia Appendix 8). Similarly, interview respondents consistently praised the Today’s Information feature for being interesting, useful, and time-appropriate. Indeed, several respondents remarked on the seemingly uncanny timing of the messages.

It’s reliable – like the people who are writing it are knowledgeable. Today’s message is always more or less in line with what I am thinking.

Baby 16 weeks, age 30-34 years, White other

I’m not the best at researching stuff and reading a big book in advance so this gives me nice little bite-size chunks of things I need to know.

Pregnant 16 weeks, age 40-44 years, White British

Other features were helping a smaller number of Baby Buddy users at the moment. This could be explained by the finding that interview respondents frequently reported that they rarely explored the app beyond the Today’s Information section. For those who did, the videos were singled out as being very helpful. Typically, respondents had viewed videos that had been highlighted in Today’s Information. Fewer respondents had explored the videos by category, although those who had appreciated the number and variety of videos available.

The information is so useful – I share it with my husband. We’ve looked at the videos – the baby moving in the womb. It’s really good for my husband because he didn’t get to go to the scan and I worry he is not bonding with the bump. He hasn’t heard the heartbeat.

Pregnant 21 weeks, age 21-24 years, Black, Asian, minority ethnicity

I like to see the videos – they’ve helped a lot to see that other women are struggling too.

Baby 16 weeks, age 30-34 years, White other

Other app features specifically praised by interview respondents included Ask Me, Your Appointments, You Can Do It, and You and Your Partner. Only one respondent was aware of Get Help. The idea of a 24/7 text helpline was widely appreciated; however, Get Help was not necessarily felt to be a good descriptor of this feature.

I love the partner’s advice. A lot of apps don’t bring the partner into it…it’s brilliant. It’s (pregnancy) a stressful time and two people don’t always react the same.

Pregnant 8 weeks, age 21-24 years, White British

The main criticism from the 10.1% of users (44/436) who felt that Baby Buddy was not helping them during the pandemic was its slowness to adapt to the new situation. Recommending courses of action that were no longer available under current circumstances, such as ask your health visitor, join a play group, and have a baby shower, were both unhelpful and a sad reminder of activities that respondents were missing out on.

One of the things that came through was a video saying keep your distance from people with colds and ‘flu and there was this pregnant woman sitting on a bench next to a man coughing. I laughed at it, but not to acknowledge when we are in a middle of a pandemic is a bit of a blunder I would say.

Pregnant 16 weeks, age 40-44 years, White British

Sometimes I won’t check because it reminds me of all the things I’d like to be doing but can’t because of social distancing.

Free text

Views on whether Baby Buddy should be providing COVID-19–specific information were mixed. While some respondents felt that this information should be prominent within the app, others expressed a keen desire that Baby Buddy remain a safe haven from the pandemic. Clarity around the guidelines and links to websites providing recommendations and advice were generally thought to be helpful.

Several respondents identified additional information and advice needs as a consequence of the pandemic. This included how to ensure your baby is gaining the right amount of weight and meeting developmental milestones (in the absence of health visitors); ideas for stimulating and entertaining babies (in the absence of classes); ways to calm a crying or fractious baby; ways to reduce one’s own anxiety and stay calm; and making sure you are getting enough vitamin D with limited time outdoors.

Things to do with him when you are stuck inside – keeping him entertained, what’s best for his development.

Baby 5 months, age 25-29 years, White British

An additional suggestion was the inclusion of self-made videos from Baby Buddy users with their tips for coping with pregnancy, birth, and parenting a very young baby during the pandemic.

Beyond content relating to the current circumstances, another notable demand for additional information came from respondents who had one or more other children. These respondents were keen to receive advice specific to their circumstances, such as how to cope with tiredness in pregnancy when you have an active toddler, how to prepare a child for the birth of their sibling, and how to avoid sibling rivalry.

The thing that I would find even more useful would be if they designed a section for 2nd or 3rd time mums…how to explain to your kids they’ve got another brother or sister coming, things like that….that would be amazing.

Pregnant 34 weeks, age 30-34 years, White other

First-time mothers suggested adding new functionalities, usually those they had found in other parenting apps. These included features to allow mothers to record feeding information (eg, time and length of feed, which breast) or diaper records and chat rooms for parents to share tips and provide mutual support.

## Discussion

### Principal Findings

This service evaluation study aimed to understand the attitudes, experiences, and needs of Baby Buddy app users in the United Kingdom during the COVID-19 pandemic, with a view to identifying ways in which Baby Buddy could further support its users. The web-based survey in conjunction with the follow-up telephone interviews revealed heightened levels of anxiety and stress around being pregnant, giving birth, and parenting a new baby during the pandemic. Reduced support, especially from health care professionals, and the impact of the lockdown clearly affected the mental and physical well-being of pregnant women and parents of young babies. Baby Buddy users appreciated having access to a source of easily accessible, reliable, and trustworthy advice, and some users felt that the app helped to compensate for the loss of face-to-face advice and support. A small minority felt that greater speed in adapting the content to reflect the new situation would have been beneficial.

Reports of insufficient support for new parents in the United Kingdom with health and parenting issues have been reported elsewhere [13]. This research showed that with more restricted access to health care services and support, accessing reliable and trustworthy information has become a key concern for pregnant women and those with young babies. Previous research has highlighted concerns women have about the reliability of pregnancy and parenting advice on the web [14] as well as the importance of trustworthiness in health apps in general [15] and pregnancy apps in particular [16]. As an NHS-approved app, and one that is often recommended to users by their health care professionals, Baby Buddy is well positioned to meet this increased demand for digital pregnancy and parenting support.

The heightened levels of anxiety and stress during the COVID-19 pandemic found among Baby Buddy users are consistent with research among the general UK population [17] and perinatal populations from other countries [18]. A study of over 4000 pregnant women in China reported a significantly higher rate of depressive symptoms (measured on the Edinburgh Postnatal Depression Scale), suggesting an increased risk of mental illness, including self-harm behaviors [19]. Studies in Turkey [20] and Canada [21,22] have also found higher rates of anxiety and depression in pregnant women during the pandemic and called for clinical surveillance and psychosocial support to mitigate adverse impacts on mothers and babies. One study was able to compare levels of anxiety and stress in the same pregnant women before and during the pandemic, and it was found that the COVID-19 pandemic is responsible for significant increases in stress [23]. There is an established body of evidence that anxiety and stress in pregnancy are risk factors for adverse outcomes for mother and baby, including longer term cognitive, emotional, and behavioral development issues in childhood [24]. Studies have indicated negative affect to be associated with preterm birth and low birth weight [24], although some surprising data are emerging showing dramatic reductions in preterm births from January to April 2020 compared to previous years [25,26]. Maternal anxiety and stress has also been shown to predict postnatal depression, potentially leading to impaired parenting quality and effectiveness [27,28]. Although face-to-face care from maternity and mental health services is clearly the optimum solution, Baby Buddy is in a position to provide support to expectant and new parents through its existing content, including the perinatal mental health films, the feature for couples (You and Your Partner), the Get Help feature, signposting to many other charities, and the Baby Buddy Crisis Messenger for 24/7 confidential and clinically assured support. The potential role of web-based resources in ameliorating COVID-19–related anxieties in perinatal women is echoed in a study among obstetricians in India [29].

This study also found physical health to be an important concern, especially among pregnant women. With lockdown limiting women’s ability to exercise, restricting their access to healthy foods, and leading to more snacking on foods high in fat, sugar, and salt, there may be a long-term impact on mothers and babies as a result of increased risks associated with excessive gestational weight gain [30,31]. These findings are consistent with data from the general UK population [32,33]. Once again, Baby Buddy can support its users by directing them to existing film and written content on exercising at home and healthy meal and snacking ideas.

The vast majority (90%) of Baby Buddy users in this study reported finding the app helpful during this period, especially given the reduced support from health care professionals, family, and friends. However, while Baby Buddy provides a wealth of information to support its users, many users were not finding all this information. It is especially important during these times of reduced support and heightened anxiety to actively encourage Baby Buddy users to explore beyond the daily information feature, Today’s Information, to find additional content and functionality. The addition of new video content featuring expectant and new parents during the COVID-19 pandemic may help to support users by normalizing feelings and experiences they are having during these unprecedented times. It may also help reduce feelings of “missing out” by exemplifying that many other young families are facing the same challenges.

With the pandemic limiting access to their usual support networks and health care professionals, Baby Buddy users have had to become more self-reliant in their roles of parenting a young baby. This has created additional information needs, particularly around baby stimulation, socialization, growth, and development. Although the app already contains written and film content covering these issues, adding new content to further meet these needs could improve the support Baby Buddy provides to its users and help increase new parents’ confidence in monitoring their baby’s well-being and development while reduced access to professional support continues.

This study was a precursor to a UK-wide survey of expectant parents and parents of children under 2 years of age, Babies in Lockdown, commissioned by Best Beginnings, Home-Start UK, and the Parent-Infant Foundation and undertaken by Critical Research [10]. The findings of the two studies are consistent and aligned. With a sample size of over 5000 and more demographic data, Babies in Lockdown was able to identify differences in response according to age, income, and ethnicity. This survey concluded that families already at risk (lower income, younger age, and from Black, Asian, minority ethnicity communities) have suffered the most as a result of the pandemic and the associated lockdown. Previous research has reported that the COVID-19 pandemic may be accentuating inequalities [34]. With one of Baby Buddy’s strengths being its disproportionately high usage by Black, Asian, minority ethnicity, and ESL parents and Best Beginnings’ remit of reducing inequalities in child health care, the Baby Buddy app has an important and pertinent role to play as the COVID-19 pandemic continues.

### Strengths and Limitations

A strength of this study was that it was conducted during the height of the COVID-19 pandemic, with data collection beginning within three weeks of the start of lockdown in the United Kingdom. As a mixed methods study, the research was able to deliver statistically robust findings alongside detailed qualitative insights.

A limitation of this study is because the survey focused on Baby Buddy users, the findings cannot be generalized to all pregnant and new parents in the United Kingdom, although its findings were highly consistent with the follow-up survey described above [10]. A further limitation of this study is self-selection bias, as respondents chose whether to respond to the survey and take part in a telephone interview. Moreover, anxiety, stress, and depression levels were self-reported rather than being measured using validated scales. A final limitation of this study was the decision to collect only age and language demographics in the web-based survey. At the time the study was designed, there was no awareness of the disproportionate risk of poor COVID-19 outcomes among Black, Asian, and minority ethnicity communities [35], and the main consideration was to reduce barriers to completion. This omission was amended for the telephone interviews and in the follow-up study referred to previously [10].

### Conclusion

The COVID-19 pandemic has created heightened anxiety and stress among expectant parents and those with a young baby. This study found that for many expectant and new parents, lockdown has had an adverse impact on their physical and mental well-being. With reductions in health and social supports, expectant and new parents are relying even more on web-based resources. As a free, evidence-based app, Baby Buddy is well positioned to meet this need. The app could support its users even more by actively directing them to the wealth of existing content relevant to their concerns and by adding content to give users the knowledge and confidence to meet the new challenges facing them. As the longer-term impact of the COVID-19 pandemic becomes apparent, it will be necessary to add to and update the content of the Baby Buddy app to meet new needs.

## Appendix

Multimedia Appendix 1 Baby Buddy Features.

Multimedia Appendix 2 Topic Guides.

Multimedia Appendix 3 Recruitment message.

Multimedia Appendix 4 Coding Framework.

Multimedia Appendix 5 Theme definitions.

Multimedia Appendix 6 Respondents’ main concerns right now.

Multimedia Appendix 7 Healthcare issues worrying respondent more than usual.

Multimedia Appendix 8 Baby Buddy features.

## Abbreviations

ESL: English as a second language
FGR: fetal growth restriction
GDPR: General Data Protection Regulation
GP: general practitioner
NHS: National Health Service
OCD: obsessive-compulsive disorder
UCL: University College London
