# Supplementary material for: Experiences, Attitudes, and Needs of Users of a Pregnancy and Parenting App (Baby Buddy) During the COVID-19 Pandemic: Mixed Methods Study
Source: JMIR Mhealth Uhealth. 2020 Dec 9;8(12):e23157. doi: 10.2196/23157 (PMC7732354; doi:10.2196/23157)
Supplement: Multimedia Appendix 1 [file mhealth_v8i12e23157_app1.pptx]

## Slide 1
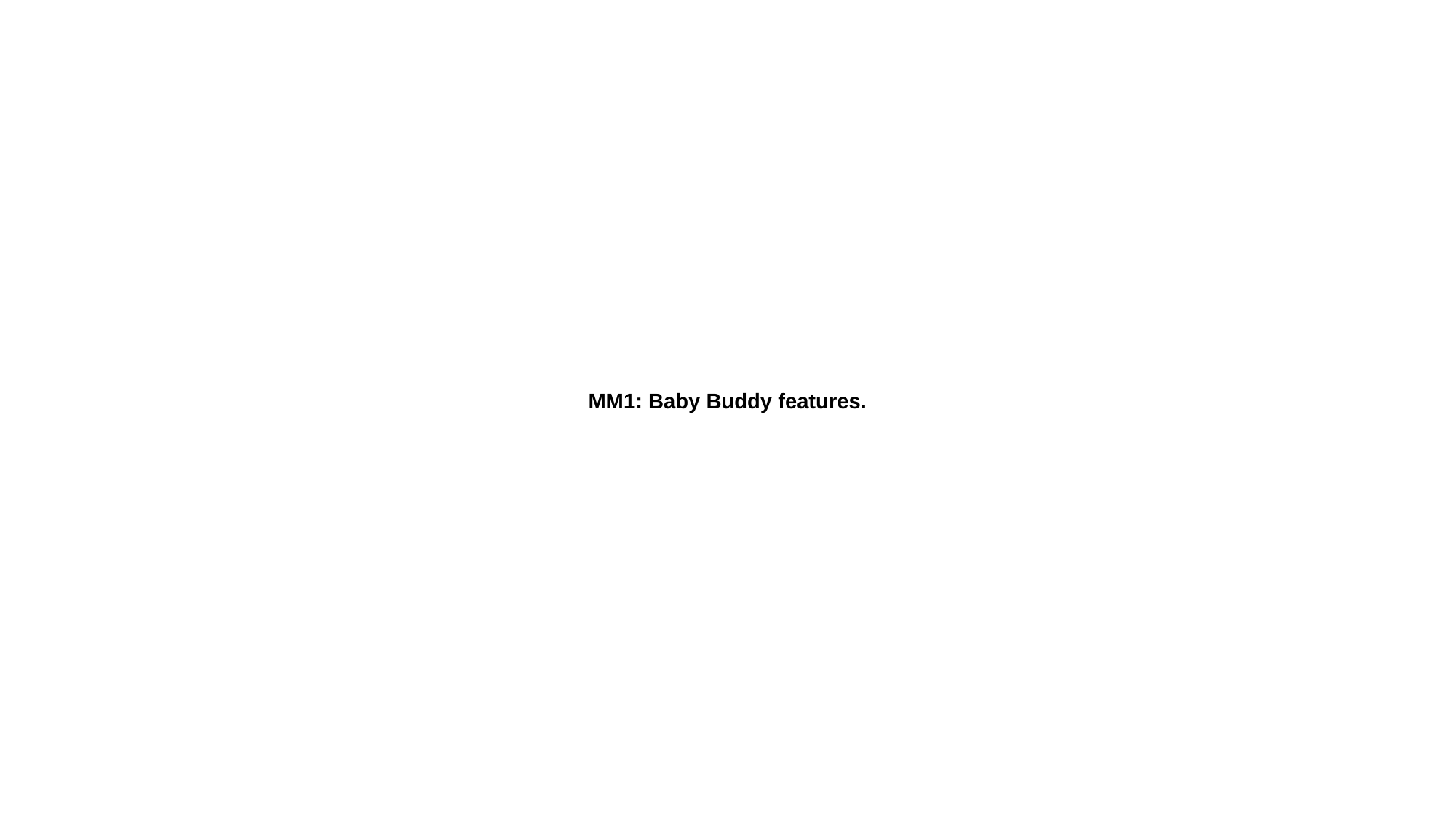

# MM1: Baby Buddy features.

## Slide 2
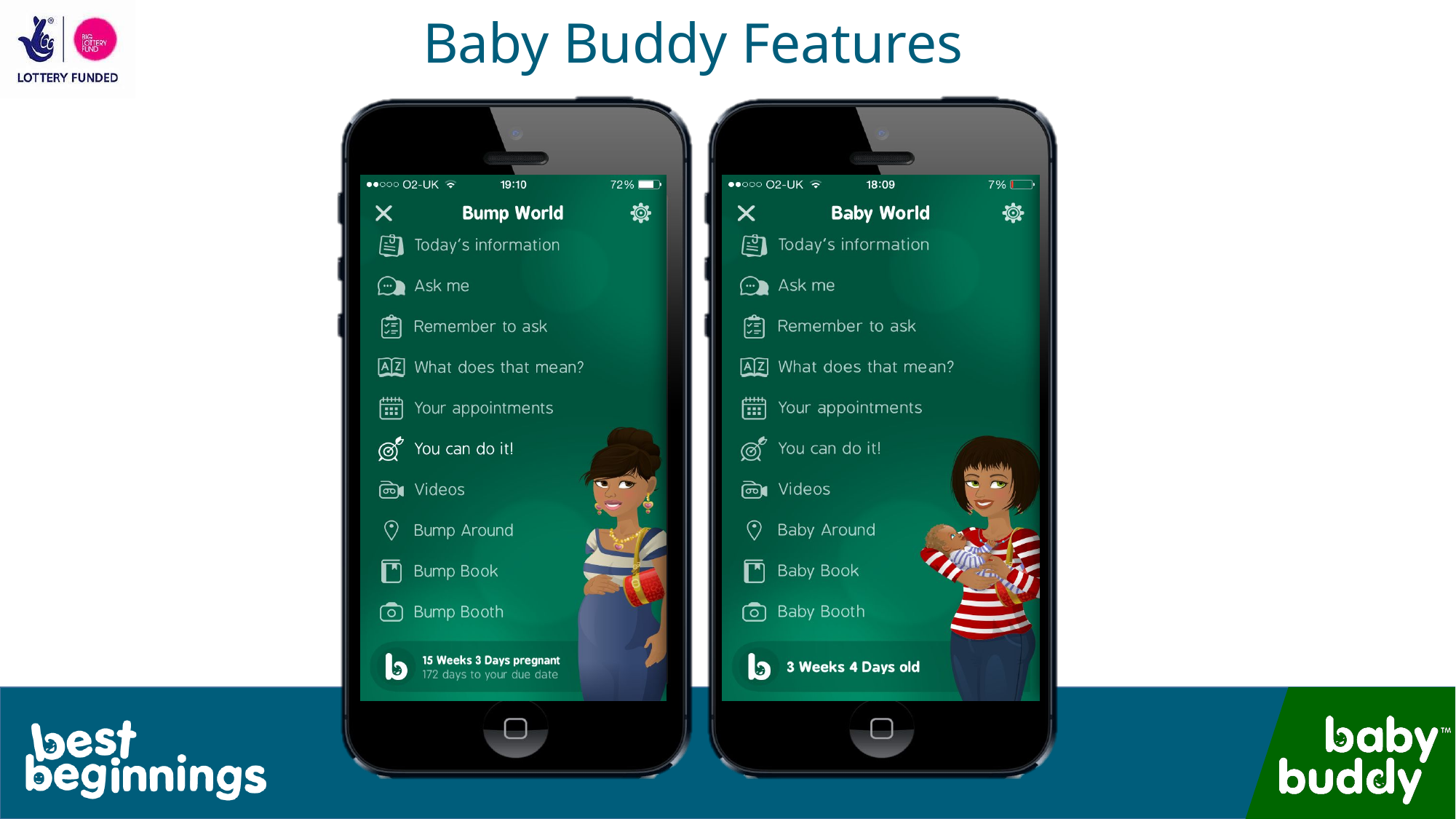

# Baby Buddy Features

## Slide 3
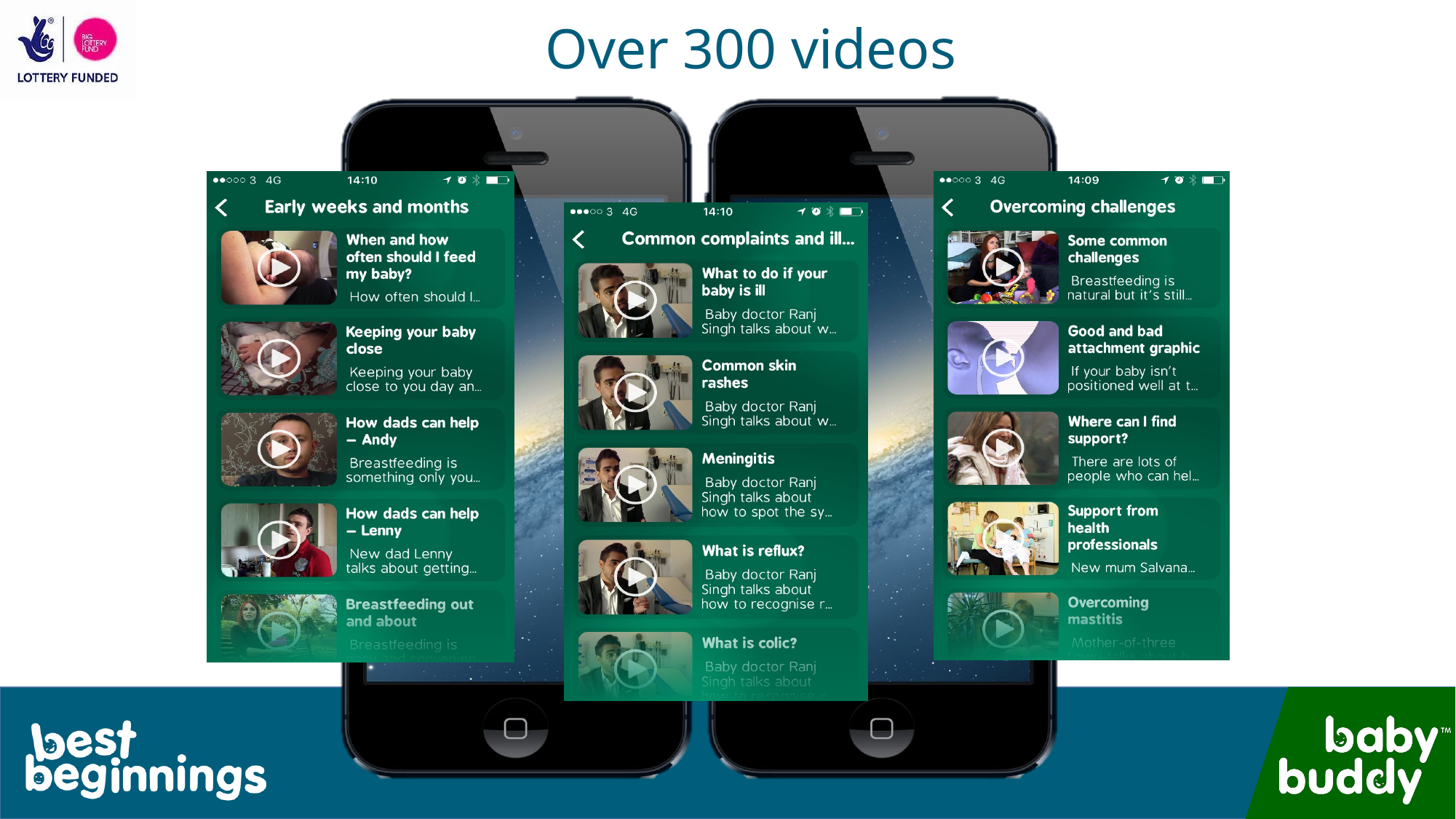

# Over 300 videos

## Slide 4
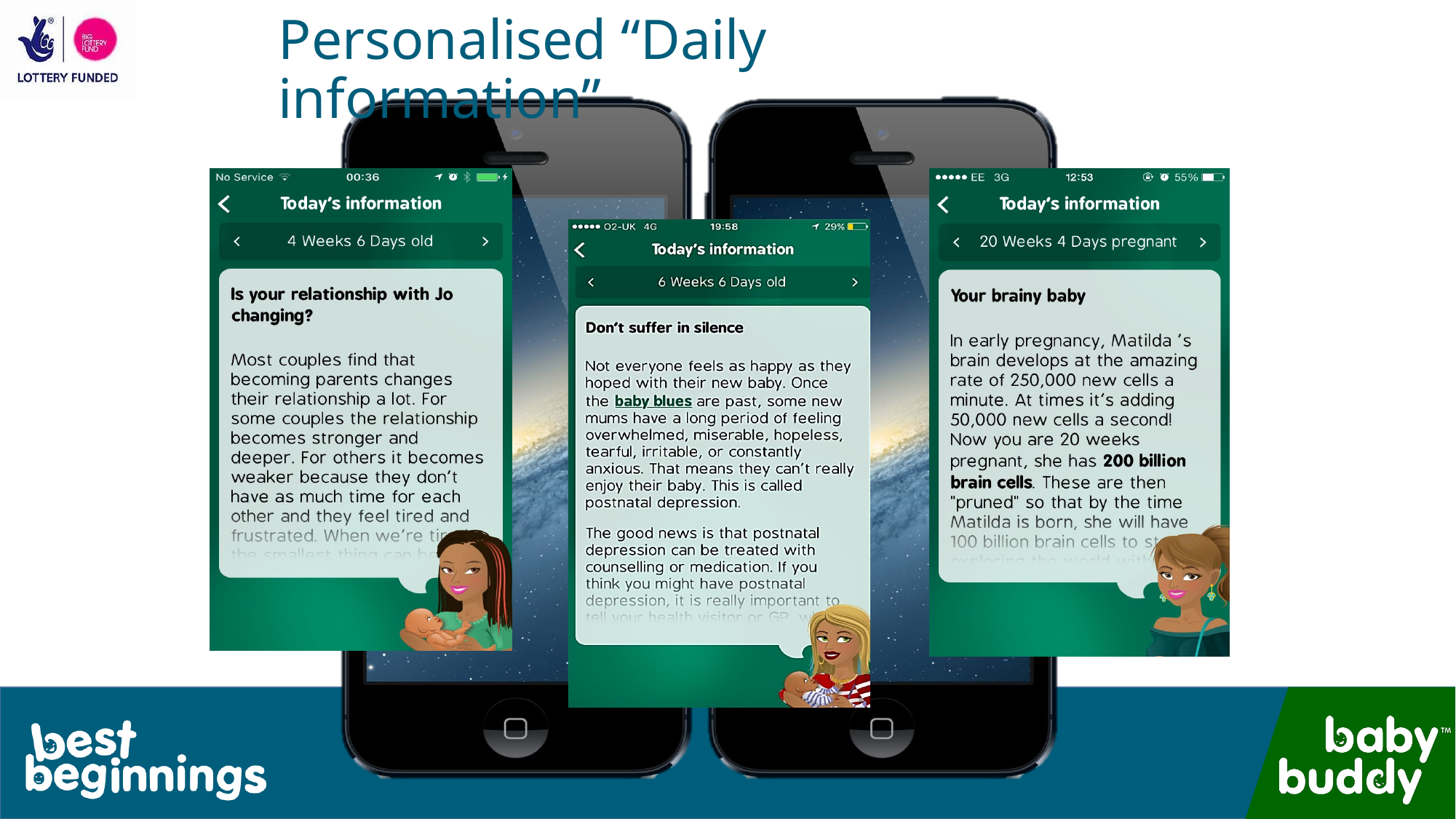

Personalised “Daily information”

## Slide 5
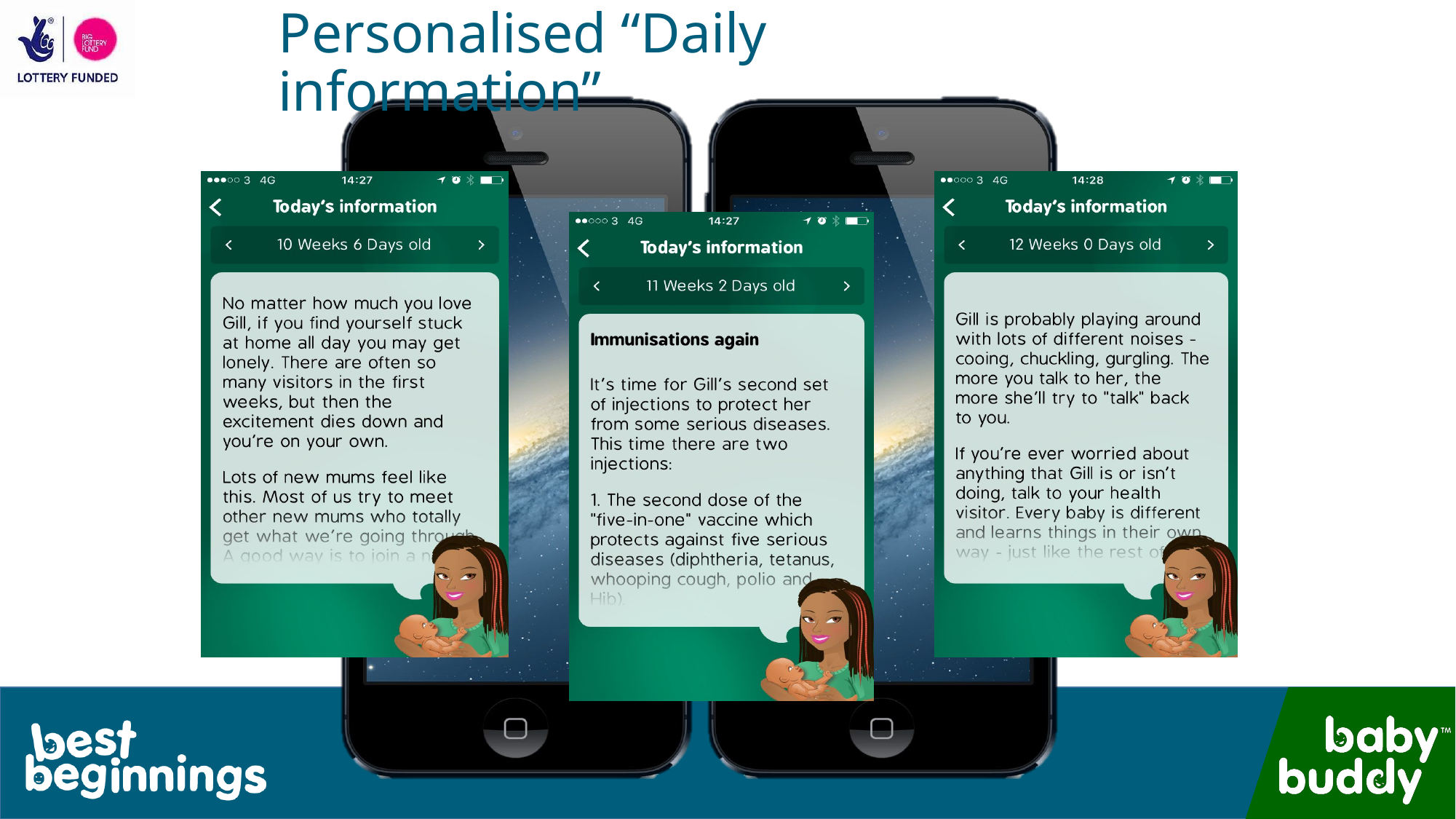

Personalised “Daily information”
