# Supplementary material for: Experiences, Attitudes, and Needs of Users of a Pregnancy and Parenting App (Baby Buddy) During the COVID-19 Pandemic: Mixed Methods Study
Source: JMIR Mhealth Uhealth. 2020 Dec 9;8(12):e23157. doi: 10.2196/23157 (PMC7732354; doi:10.2196/23157)
Supplement: Multimedia Appendix 2 [file mhealth_v8i12e23157_app2.docx]

**MM2: Topic Guides.**

Telephone interview topic guide: Expectant parents

Thank you for taking part in this telephone interview. We want to assure you that we shall be respecting your privacy and complying with GDPR. The findings from this research will be published on the Best Beginnings website and possibly in the academic press, but we will never reveal your identity and we shall ensure that your contact details are not linked to any records of this interview. You can terminate this interview at any stage and decline to answer questions if you want. If you change your mind about taking part, you have the option of emailing us to withdraw your interview for up to a week from today. After this time, we shall delete your contact details from our records so we shall no longer be able to delete your interview. We would also like your permission for us to tape record this interview. If we have your consent to proceed on this basis, let's get started.

- How has your pregnancy been so far?
- What aspects were you enjoying  the most?
- What aspects have been more challenging?
- Do you feel been pregnant pre-Coronavirus was very different to being pregnant now? (If pregnant before CV)
- How have you been feeling about your pregnancy during this time of coronavirus?
  - What are the biggest concerns you have right now (probe access to food; financial worries and their impact; housing issues; relationship issues)?
  - Has Coronavirus affected your mental well-being/mood/your thoughts? If so how?
  - Has Coronavirus affected your physical health (probe diet, physically activity)? If so how?
- How  well-informed do you feel about what you as an expectant parent should be doing during Coronavirus?
  - Are you getting access to the information you need/are looking for?
  - Are you aware of Government recommendations for pregnant women; if so, what do you think about these; if not what do you think you should be doing?
- Do you feel motivated to follow the Government advice; why/why not?
  - Has stay at home impacted you? If so how?
  - How are relationships with partner/others in the household?
  - Are you or anyone in your house going out to work/a key worker/healthcare worker? If so, how is this affecting you.
  - Are you working? If so, how do you feel about that?
  - Are you going out to the shops/for walks? How do you feel about that?
- Has coronavirus affected your ante-natal care?
  - Are you still going to appointments, if not, why not (probe concerns about safety); if so where; have any of your appointments been telephone or video call; if so what has your experience of these been; have you had any email/text communication with your healthcare professionals; if so what has your experience of this been?
  - Overall do you feel happy about your ante-natal care; why/why not?
- Has Coronavirus made you feel differently about giving birth and being a parent/having a new baby? If so how.
- What are you most excited about?
- What are you most concerned about? (probe the birth, baby’s risk of Coronavirus, access to baby formula; postnatal care; sharing the occasion with wider family and friends etc)
- Do you think Coronavirus will affect your baby in any way? If your baby could speak what do you think his/her opinion would be?
- Do you feel you are getting enough support through this Coronavirus?
  - If so who is supporting you and how?
  - If not, why not and what kind of support do you feel you are lacking?
- Has Coronavirus affected your sources of advice on pregnancy/parenthood matters?
  - What, if any, online sources have you been using?
- Tell us about you and Baby Buddy
  - How did you first come across Baby Buddy?
  - How often do you use it; what you like about it; which features do you use?
- Has the way in which you use Baby Buddy changed since coronavirus? If so how?
- Which, if any, of the features are helping you at the moment?
- What information would you like Baby Buddy to focus on more?
- How can Baby Buddy further support you during this time?
- Probe areas such as partner conflict, crisis text messenger etc ?
- What aspects would you like to be added onto the App?
- Would you like Baby Buddy to introduce relevant news (regarding Coronavirus and pregnancy)? Why or why not?

Thank you for taking part in this interview, do you have anything you would like to add or ask?

Telephone interview topic guide: New parents

Thank you for taking part in this telephone interview. We want to assure you that we shall be respecting your privacy and complying with GDPR. The findings from this research will be published on the Best Beginnings website and possibly in the academic press, but we will never reveal you identity and we shall ensure that your contact details are not linked to any records of this interview. You can stop this interview at any stage and decline to answer questions if you want. If you change your mind about taking part, you have the option of emailing us to withdraw your interview for up to a week from today. After this time, we shall delete your contact details from our records so we shall no longer be able to delete your interview. We would also like your permission for us to tape record this interview. If we have your consent to proceed on this basis, let's get started.

- When was your baby born? (Questions will be adjusted according to whether baby was born before or after lockdown/March 23rd)
- How would you describe the pregnancy pre-Coronavirus?
- What aspects were you enjoying  the most?
- What aspects were more challenging?
- How have you been feeling about being a parent to a young baby during this time of coronavirus?
  - What are the biggest concerns you have right now (probe access to food; financial worries and their impact; housing issues; relationship issues)?
  - Has Coronavirus affected your mental well-being/mood/your thoughts? If so how?
  - Has Coronavirus affected your physical health (probe diet, physically activity)? If so how?
  - Are you or anyone in your house going out to work/a key worker/healthcare worker? If so, how is this affecting you?

**If birth was after March 23^rd^**

- How was it being pregnant during lockdown?
  - How  well-informed did you feel about what you as an expectant parent should be doing during Coronavirus?
  - Did you getting access to the information you needed?
  - Were you aware of Government recommendations for pregnant women; if so, what do you think about these; if not what do you think you should be doing; did you go out to work/to the shops/for walks/exercise?
  - Did you feel motivated to follow the Government advice; why/why not?
- Tell us about the birth and how that was for you (and your partner)?
  - Did it go according to how you had planned it?
  - How do you feel Coronavirus affected your birth experience?

**All:**

- How  well-informed do you feel about what you as a parent of a young baby should be doing during Coronavirus?
  - Are you getting access to the information you need/are looking for?
  - Are you aware of Government recommendations; if so, what do you think about these; if not what do you think you should be doing?
  - Do you feel motivated to follow the Government advice; why/why not?
- How has stay at home impacted you?
  - How are relationships with partner/others in the household?
  - Are you or anyone in your house going out to work/a key worker/healthcare worker? If so, how is this affecting you.
  - Are you working? If so, how do you feel about that?
  - Are you going out to the shops/for walks? How do you feel about that?
- How has coronavirus affected your postnatal care (NB explore visits to GPs surgeries and health visitors for baby appointments)?
  - Are you still going to appointments, if not, why not; if so where; have any of your appointments been telephone or video call; if so what has your experience of these been; have you had any email/text communication with your healthcare professionals; if so what has your experience of this been?
  - Overall do you feel happy about your postnatal care; why/why not?
- Has coronavirus affected your feelings about being a parent to a new/young baby? If so how?
  - What are you most concerned about? (probe baby’s risk of Coronavirus, access to baby formula; postnatal care; social isolation/ not having contact with wider family and friends etc)
- Do you think Coronavirus will affect your baby in any way? If your baby could speak what do you think his/her opinion would be?
- Do you feel you are getting enough support through this Coronavirus?
  - Who is supporting you and how?
  - If not, why not and what kind of support do you feel you are lacking?
- Has Coronavirus affected your sources of advice on baby/parent matters?
  - What, if any, online sources have you been using?
- Tell us about you and Baby Buddy
  - How did you first come across Baby Buddy
  - How often do you use it; what you like about it; which features do you use?
- Has the way in which you use Baby Buddy changed since Coronavirus ?
- Which features are helping you at the moment?
- What information would you like Baby Buddy to focus on more?
- How can Baby Buddy further support you during this time?
- Probe areas such as partner conflict, crisis text messenger etc ?
- What aspects would you like to be added onto the App?
- Would you like Baby Buddy to introduce relevant news (regarding Coronavirus and pregnancy)? Why or why not?

Thank you for taking part in this interview, do you have anything you would like to add or ask?
