# Supplementary material for: Experiences, Attitudes, and Needs of Users of a Pregnancy and Parenting App (Baby Buddy) During the COVID-19 Pandemic: Mixed Methods Study
Source: JMIR Mhealth Uhealth. 2020 Dec 9;8(12):e23157. doi: 10.2196/23157 (PMC7732354; doi:10.2196/23157)
Supplement: Multimedia Appendix 3 [file mhealth_v8i12e23157_app3.docx]

**MM3: Recruitment message.**

“Is Baby Buddy supporting you right now? Please complete this short survey
<https://www.surveymonkey.co.uk/r/BabyBuddy> Thank you in advance - we really value your feedback”
