# Supplementary material for: Experiences, Attitudes, and Needs of Users of a Pregnancy and Parenting App (Baby Buddy) During the COVID-19 Pandemic: Mixed Methods Study
Source: JMIR Mhealth Uhealth. 2020 Dec 9;8(12):e23157. doi: 10.2196/23157 (PMC7732354; doi:10.2196/23157)
Supplement: Multimedia Appendix 4 [file mhealth_v8i12e23157_app4.docx]

**MM4: Coding Framework.**

**Initial Coding Framework.**

1. **Influences and outcomes of the pandemic on my wellbeing**

| Influence  Outcomes | COVID 19 | Stay at home and other Government guidelines | Healthcare Changes;  antenatal, postnatal and other |
| --- | --- | --- | --- |
| My mental health | **1 a** | **1b** | **1c** |
| My physical health | **1d** | **1e** | **1f** |
| My partnership/our relationship | **1g** | **1h** | **1i** |
| My baby | **1j** | **1k** | **1l** |
| My/our future | **1m** | **1n** | **1o** |

1. **Variables affecting influences and outcomes**

**2a** My work situation

**2b** My partner’s work situation

**2c** Housing conditions

**2d** Financial situation

**2e** Support networks

**2f** My physical and/or mental vulnerabilities

**2g** My partner’s physical and/or mental vulnerabilities

**2h** Gestational stage/baby’s age

**2i** Other (including ethnicity, ESL, age

1. **Sources of information, advice and support at this time**

**3a** Current sources of information, advice and support

**3a** Changes in sources of information, advice and support

**3c** Apps, online and sources being used at this time

**3d** Other information, advice and support issues

1. **Baby Buddy**

**4a** Usage of Baby Buddy at this time

**4b** Changes in usage of Baby Buddy

**4c** Ways in which Baby Buddy is helping at this time

**4d** Shortcomings of Baby Buddy at this time

**4e** Ideas for improvements to Baby Buddy
