# Supplementary material for: Experiences, Attitudes, and Needs of Users of a Pregnancy and Parenting App (Baby Buddy) During the COVID-19 Pandemic: Mixed Methods Study
Source: JMIR Mhealth Uhealth. 2020 Dec 9;8(12):e23157. doi: 10.2196/23157 (PMC7732354; doi:10.2196/23157)
Supplement: Multimedia Appendix 5 [file mhealth_v8i12e23157_app5.docx]

**MM5: Theme Definitions.**

**Theme Definitions and Examples.**

| **Theme** | **Definition** | **Examples** |
| --- | --- | --- |
| 1. **Causes of increased levels of anxiety** | Any changes in levels of anxiety, stress or other negative states as a consequence of COVID-19 | See below |
| - 1. Current disruption | Disruption to all aspects of respondent’s life caused by COVID-19 | Food shortages; antenatal appointments cancelled; play groups cancelled; no family/informal childcare support |
| - 1. Future uncertainties | Worries about the effect of COVID-19 on all aspects of respondent’s future | Effect on birth plan; timing of return to work; long term effects on baby’s development; securing childminder/nursery school |
| - 1. Fear of catching COVID-19 | Fears relating to their own/partner’s susceptibility to COVID-19 and the consequences of either being ill | Transfer to baby; childcare issues; caring for sick partner and a new baby |
| 1. **Reduced levels of support** | Changes as a result of COVID-19 to all aspects of support respondents receive in the context of pregnancy, birth and parenting | See below |
| - 1. Support from friends and family | Changes in contact with and support from friends and family | Zoom only contact; new babies not meeting grandparents; no face-to-face antenatal groups |
| - 1. Support from HCPs | Changes in support from GPs, midwives, health visitors and maternity units | Effect of loss of/reduced health visitor services; examples of physical consequences of no/telephone only appointments |
| - 1. Government support | Communications from Government about advice for expectant and new parents | Changing advice re shielding; little/no advice on babies |
| 1. **Life under lockdown** | Any changes to lifestyle, mental and physical well-being, relationships as a result of lockdown | See below |
| - 1. Missing out | Effect of lockdown on social life |  |
| - 1. Lifestyle disruption | Effect of lockdown of diet and exercise | Reduced opportunities to exercise; increased snacking; changes to shopping behaviour |
| - 1. Couple relationships | Effect of lockdown on couple’s relationship | Benefits and downsides of partners working from home; conflict over hygiene |
| - 1. Impact on baby | Effect of lockdown on baby | Fewer opportunities for socialisation; disrupted/better routines |
| 1. **Baby Buddy** | All comments relating to Baby Buddy | See below |
| - 1. Baby Buddy usage | Current usage and how this might be different from non-COVID-19 times | Turning to online resources as face-to-face become scarce |
| - 1. Baby Buddy strengths and weaknesses | How Baby Buddy is helping/not helping during COVID-19 times | Inappropriate advice in current climate; relevant bite size information |
| - 1. New ideas for Baby Buddy | How Baby Buddy could be improved, generally and specifically for COVID-19 times | Peer group videos to normalise anxieties; ideas for baby stimulation; developmental milestone |
