# Supplementary material for: Experiences, Attitudes, and Needs of Users of a Pregnancy and Parenting App (Baby Buddy) During the COVID-19 Pandemic: Mixed Methods Study
Source: JMIR Mhealth Uhealth. 2020 Dec 9;8(12):e23157. doi: 10.2196/23157 (PMC7732354; doi:10.2196/23157)
Supplement: Multimedia Appendix 6 [file mhealth_v8i12e23157_app6.docx]

| **Concerns** | **Pregnant (n=235)** | **Postnatal (n=188)** | **Total**  **(n=423)** |
| --- | --- | --- | --- |
| My baby’s health | Not asked | 63% (119) | 63% (119) |
| My emotional and mental health | 61% (144) | 60% (109) | 60% (253) |
| Getting reliable pregnancy information and advice | 46% (109) | 49% (92) | 48% (201) |
| My physical health | 45% (106) | 35% (65) | 40% (171) |
| Eating a healthy diet | 35% (83) | 28% (53) | 32% (136) |
| Financial worries | 28% (65) | 21% (39) | 25% (104) |
| My relationship with my partner and/or others in my household | 19% (45) | 31% (58) | 24% (103) |
| Looking after my partner’s mental health | 10% (24) | 19% (36) | 14% (60) |
| Getting the medicines I need | 10% (23) | 13% (24) | 11% (47) |
| My partner’s physical health | 7% (16) | 11% (20) | 9% (46) |
| Housing issues | 10% (23) | 6% (12) | 8% (35) |
| None of the above | 9% (22) | 11% (20) | 10% (42) |

**MM6**: **Respondents’ main concerns right now.**

Question asked: What are your main concerns right now? (Tick all that apply to you)
