# Supplementary material for: Experiences, Attitudes, and Needs of Users of a Pregnancy and Parenting App (Baby Buddy) During the COVID-19 Pandemic: Mixed Methods Study
Source: JMIR Mhealth Uhealth. 2020 Dec 9;8(12):e23157. doi: 10.2196/23157 (PMC7732354; doi:10.2196/23157)
Supplement: Multimedia Appendix 7 [file mhealth_v8i12e23157_app7.docx]

|  |  | **Pregnant (n=235)** |  |  | **Postnatal (n=188)** |  |
| --- | --- | --- | --- | --- | --- | --- |
| **Concerns** | Overall | Weeks 1-2  (n=113) | Weeks 6-7  (n=38) | Overall | Weeks 1-2  (n=74) | Weeks 6-7  (n=42) |
| Staying safe when giving birth | 65% (152) | 69% (78) | 58% (22) |  | Not asked |  |
| Seeing my doctor (midwife) or health visitor if I need to | 53% (125) | 58% (65) | 39% (15) | 59% (110) | 59% (44) | 57% (24) |
| Caring for a new baby under the ‘stay at home advice’ | 46% (109) | 41% (46) | 55% (21) | 49% (92) | 43% (32) | 55% (23) |
| Staying safe at my antenatal appointments | 51% (120) | 55% (62) | 45% (17) |  | Not asked |  |
| Staying safe at my postnatal or baby appointments |  | Not asked |  | 38% (71) | 42% (31) | 33% (14) |
| Getting to my antenatal appointments | 31% (73) | 36% (41) | 21% (8) |  | Not asked |  |
| Getting to my postnatal or baby appointments |  | Not asked |  | 35% (48) | 27% (20) | 19% (8) |

**MM7: Healthcare issues worrying respondent more than usual.**

**(comparing first two weeks of survey with last two weeks).**

Question asked: Are any of the following worrying you more than usual? (Tick all that apply to you)
