# Supplementary material for: Experiences, Attitudes, and Needs of Users of a Pregnancy and Parenting App (Baby Buddy) During the COVID-19 Pandemic: Mixed Methods Study
Source: JMIR Mhealth Uhealth. 2020 Dec 9;8(12):e23157. doi: 10.2196/23157 (PMC7732354; doi:10.2196/23157)
Supplement: Multimedia Appendix 8 [file mhealth_v8i12e23157_app8.docx]

| **Features** | **Pregnant (n=211)** | **Postnatal (n=177)** | **Total (n=388)** |
| --- | --- | --- | --- |
| Today’s information | 97% (205) | 97% (172) | 97% (377) |
| Videos | 40% (85) | 45% (79) | 42% (164) |
| You and your partner | 14% (30) | 13% (23) | 14% (53) |
| Ask me | 10% (22) | 12% (22) | 11% (44) |
| Get help | 6% (13) | 7% (12) | 6% (25) |
| You can do it! | 3% (7) | 8% (15) | 6% (22) |

**MM8: Baby Buddy features respondents are finding most helpful at the moment.**

Question asked: Which  Baby Buddy features are you finding most helpful at the moment? (Tick all that apply to you)
